# Further Evidence of Anthropogenic Impact: High Levels of Multiple-Antimicrobial-Resistant Bacteria Found in Neritic-Stage Sea Turtles

**DOI:** 10.3390/antibiotics13110998

**Published:** 2024-10-22

**Authors:** Ming-An Tsai, I-Chun Chen, Zeng-Weng Chen, Tsung-Hsien Li

**Affiliations:** 1 Department of Veterinary Medicine, College of Veterinary Medicine, National Pingtung University of Science and Technology, Pingtung 91201, Taiwan; william878588@gmail.com; 2 International Program in Ornamental Fish Technology and Aquatic Animal Health, International College, National Pingtung University of Science and Technology, Pingtung 91201, Taiwan; 3 National Museum of Marine Biology and Aquarium, Pingtung 94450, Taiwan; bbc123@nmmba.gov.tw; 4 Animal Technology Research Center, Agricultural Technology Research Institute, Miaoli 340401, Taiwan; zwc@mail.atri.org.tw; 5 Department of Marine Biotechnology and Resources, National Sun Yat-sen University, Kaohsiung 804201, Taiwan; 6 Institute of Marine Ecology and Conservation, National Sun Yat-sen University, Kaohsiung 804201, Taiwan; 7 IUCN Species Survival Commission, Marine Turtle Specialist Group for the East Asia Region, Taiwan

**Keywords:** antimicrobials, multiple antibiotic resistance index, turtles, heavy metal resistance genes

## Abstract

Background/Objectives: Marine turtles are globally threatened and face daily anthropogenic threats, including pollution. Water pollution from emerging contaminants such as antimicrobials is a major and current environmental concern. Methods: This study investigated the phenotypic antimicrobial resistance and heavy metal resistance genes of 47 *Vibrio* isolates from different stages of sea turtles (oceanic stage vs neritic stage) from the Taiwanese coast. Results: The results show that a high proportion (48.9%; 23/47) of the *Vibrio* species isolated from sea turtles in our study had a multiple antimicrobial resistance (MAR) pattern. It was found that *Vibrio* spp. isolates with a MAR pattern and those with a MAR index value greater than 0.2 were both more likely to be observed in neritic-stage sea turtles. Furthermore, isolates from neritic-stage sea turtles exhibited greater resistance to the majority of antimicrobials tested (with the exception of beta-lactams and macrolides) than isolates from the oceanic-stage groups. Isolates from neritic sea turtles were found to be more resistant to nitrofurans and aminoglycosides than isolates from oceanic sea turtles. Furthermore, isolates with a MAR pattern (*p* = 0.010) and those with a MAR index value greater than 0.2 (*p* = 0.027) were both found to be significantly positively associated with the mercury reductase (*merA*) gene. Conclusions: The findings of our study indicate that co-selection of heavy metals and antimicrobial resistance may occur in aquatic bacteria in the coastal foraging habitats of sea turtles in Taiwan.

## 1. Introduction

A total of five sea turtle species have been recorded in Taiwan, including the green turtle (*Chelonia mydas*; endangered), hawksbill turtle (*Eretmochelys imbricata*; critically endangered), olive ridley turtle (*Lepidochelys olivacea*; vulnerable), loggerhead turtle (*Caretta caretta*; vulnerable), and leatherback turtle (*Dermochelys coriacea*; vulnerable) [1]. All of the species of sea turtles are included in the Red List of Threatened Species, which is maintained by the World Conservation Union (IUCN Red List). In addition, they are listed under the Schedule of Protected Marine Species, which is managed by the Ocean Affairs Council of the Executive Yuan in Taiwan. Nevertheless, sea turtles have been adversely affected by anthropogenic activities, including non-target bycatch, coastal development, marine debris, global environmental change, marine pollution, and anthropogenic-exacerbated diseases such as fibropapillomatosis (FP), among others [1,2,3,4,5,6,7]. As sea turtles play a pivotal role in maintaining the health of marine ecosystems [8,9], previous studies have utilised these animals as biological indicators to determine pollution levels in local marine environments [10,11,12,13,14]. Due to their high fidelity to coastal feeding grounds and nesting sites, their longevity, and their frequent use of nearshore habitats affected by anthropogenic activities, marine turtles are frequently exposed to antimicrobial-resistant bacteria, antimicrobial residues, and heavy metals. This is particularly prevalent in areas with a high level of environmental pollution [6,11,15,16,17,18].

Antimicrobials are regarded as one of the most significant emerging contaminants, due to the paucity of data regarding their cumulative toxic effects on aquatic organisms and the fact that their continued presence leads to the development of antimicrobial-resistant bacteria [19,20]. In fact, it has been demonstrated that anthropogenic activities resulting in the generation of agricultural and aquaculture wastes, in addition to water run-off, can facilitate the introduction of multiple antimicrobial resistance organisms and their associated resistance factors into the environment and water bodies [21,22,23,24]. In particular, the excessive and inappropriate use of antimicrobials (including nitrofurantoin, amikacin, and gentamicin) in human and veterinary medicine, as well as in agriculture and aquaculture, has led to an increase in selective pressure on bacteria, resulting in the emergence of antimicrobial-resistant strains and the dissemination of antimicrobial resistance throughout various environments [19,23,25,26,27,28,29,30,31,32,33,34]. Hence, the use of antimicrobials in the aquaculture industry has been prohibited or severely regulated in numerous countries across Europe and North America due to the potential for adverse effects, the presence of residues in animal tissue, the emergence of bacterial resistance, and the risk of environmental contamination [23,30,35,36]. Nevertheless, they are used in other regions, particularly in Asia [19,30,37,38,39].

Additionally, the development of bacterial resistance to antimicrobials can be influenced not only by the antimicrobials themselves but also by other pollutants, such as heavy metals. Previous studies have demonstrated a correlation between heavy metals and the selection of antimicrobial resistance genes, indicating that these pollutants can induce resistance in metal-contaminated environments [40,41,42,43,44,45]. A study by Fang et al. (2020) [46] revealed that 22.32% of the *V. parahaemolyticus* isolates identified in Pacific mackerel exhibited multi-heavy metal resistance. A study conducted in southwest Nigeria demonstrated that all *Vibrio* isolates from water in aquaculture ponds with elevated metal contamination levels exhibited tolerance to five metals (copper, zinc, lead, nickel, and chromium) [47]. It has been demonstrated that antimicrobial-resistant *Vibrio* isolates demonstrate tolerance to heavy metals, including cadmium, zinc, and copper [41,48,49]. Furthermore, in 2018 Kang et al. reported antibiotic and heavy metal resistance (Ba^2+^, Co^3+^, Cd^2+^, and Cu^2+^) of *V. ahemolyticus* (*n* = 59) isolated from oysters in Korea [50]. However, there are currently no published data on the heavy metal resistance patterns of *Vibrio* strains in sea turtles in Taiwan.

The aforementioned circumstances have the consequence that aquatic environments act as reservoirs of antimicrobial-resistant bacteria from a variety of sources. These include human wastewater, hospital effluents, and animal and plant agricultural runoff [23,24,51,52]. It is well documented that sea turtles typically spend a significant portion of their lives submerged beneath the surface of the aquatic environment, with the majority of their time spent in this state [53,54]. Furthermore, it has been demonstrated that antimicrobial-resistant bacteria have been detected in both healthy wild sea turtles and those that are injured or unwell and undergoing rehabilitation; as a result, it can be postulated that the effectiveness of clinical treatments for microbial infections in sea turtles that are rescued may be severely limited [5,6,11,18,55,56,57]. Although previous studies have indicated a potential association between antimicrobial-resistant strains derived from sea turtles and coastal effluent pollution [6,11,12,15,58], further investigation is required to elucidate this hypothesis. Because sea turtles exhibit high site fidelity throughout their various life stages (neritic and oceanic) [18,59,60,61], they are optimal candidates for sentinel programs investigating the sources of antibiotic-resistant bacteria in sea turtles in Taiwan. The early juvenile stage, which is observed in the open ocean, is referred to as the oceanic stage. In contrast, the juvenile stage that is found in coastal waters is designated as the neritic stage [18,59,60,61].

In recent decades, there has been a notable rise in antimicrobial resistance among various bacterial species, including *Vibrio*. This phenomenon may be attributed to the pervasive misuse of antimicrobials in both agricultural and human systems [20,24,50,62,63]. Vibriosis, the most prevalent bacterial disease affecting a wide range of marine and estuarine fish species, is primarily caused by *V. harveyi*, *V. parahaemolyticus*, and *Vibrio vulnificus*, which are among the most frequent fish pathogens, leading to significant economic losses in marine or estuarine aquaculture practices [24]. Furthermore, *Vibrio* species have often been found in sea turtles around the world [6,14,16,17,64]. Tsai et al. (2021) reported *Vibrio* spp. as the most dominant (31.91%) species in Taiwanese sea turtles, with *V. alginolyticus* (46.66%), *V. harveyi* (20.00%), *V. vulnificus* (20.00%), *V. cholerae* (6.66%), and *V. metschnikovii* (6.66%) being the most prevalent *Vibrio* species identified in that study [6].

It has been proposed that the assessment of antimicrobial resistance and the identification of an association between different antimicrobial resistance characteristics can prevent the further selection of resistance, thus constituting a critical tool in the development of efficient control guidelines [24,65]. Given the aforementioned circumstances, this study aimed to investigate further evidence of anthropogenic impact on sea turtles by comparing the levels of phenotypic antimicrobial resistance and heavy metal resistance genes from *Vibrio* species found in different life stages of these reptiles.

## 2. Results

### 2.1. Antimicrobial Susceptibility Test Results

**Figure 1:**
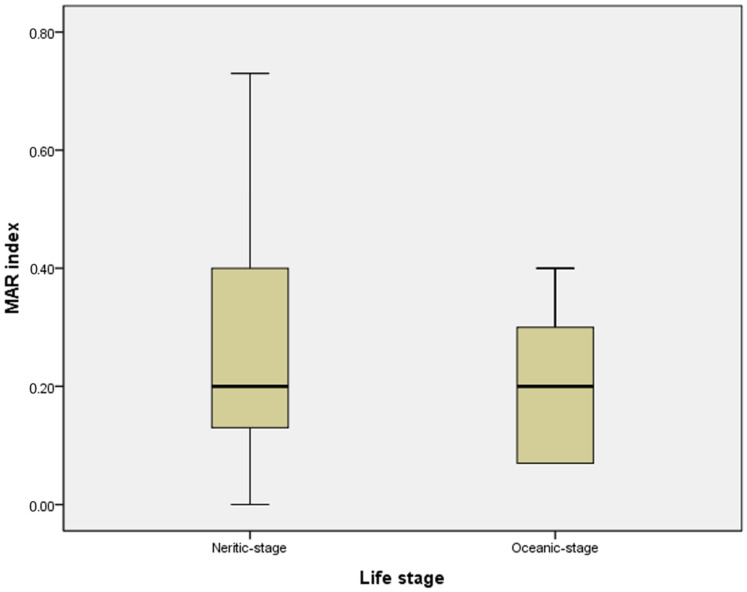
A comparative analysis of multiple antimicrobial resistance (MAR) index values for *Vibrio* spp. isolates derived from different life stages of sea turtles.

The rank order of antimicrobial resistance of the evaluated *Vibrio* spp. isolates was as follows: β-lactams (78.7%) > cephalosporins (72.3%) > aminoglycosides (29.7%) > nitrofurans (29.7%) > tetracyclines (27.6%) > fluoroquinolones (23.4%) > folate pathway inhibitors (12.7%) > macrolides (10.6%) > phenicols (4.2%). With regard to the multiple antimicrobial resistance phenotype patterns, we found that a high proportion (48.9%; 23/47) of *Vibrio* species isolated from sea turtles in our study possessed multiple antimicrobial resistance (MAR) patterns (i.e., resistance to three or more antimicrobial classes) (Table 1 and Table S1). No association was found between the source of isolation (turtle life stage, species, or stranding area) and MAR patterns in this study. However, compared to 28.6% of isolates with MAR patterns in the oceanic-stage group, a higher percentage (52.5%) of isolates with MAR patterns was observed in the neritic-stage group [Odds ratio (OR) = 2.76; 95% confidence interval (CI) = 0.48–15.95]. The MAR index of the isolates varied between 0.00 and 0.73. However, there was no significant difference in the MAR index between bacteria isolated from neritic-stage and oceanic-stage groups (Figure 1). There was no significant difference in the MAR index between the bacteria isolated from the different species of turtles. In addition, there was no significant difference in the MAR index between turtles from different stranding areas. Our study also found that 40.4% of isolates exhibited a MAR index greater than 0.2, with a higher prevalence observed in the neritic stage (42.5%) compared to the oceanic stage (28.6%) (OR = 1.85; 95% CI = 0.32–10.69). Isolates from neritic-stage sea turtles were more resistant to almost all classes of antimicrobial agents (except β-lactams and macrolides) than isolates from the oceanic-stage groups. In particular, isolates from neritic sea turtles were found to be more resistant to nitrofurans and aminoglycosides than those from oceanic sea turtles. Thus, the neritic life stage of sea turtles was identified as a potential risk factor for nitrofuran and aminoglycoside resistance, both of which were almost significantly associated (*p* = 0.086). Nevertheless, isolates from sea turtles at the oceanic stage did not show antimicrobial resistance to antimicrobial agents such as nitrofurans, folate pathway inhibitors, aminoglycosides, and phenicols (Table 2).

### 2.2. Detection of Heavy Metal Resistance Genes

Regarding the results of isolates carrying the metal resistance genes, no cadmium resistance gene (*czcA*) was detected in any of the isolates. The first, second, and third most frequently identified metal resistance genes were the arsenate reductase gene (*arsC*) (33 isolates), the chromium resistance gene (*chrA*) (32 isolates), and the mercuric reductase gene (*merA*) (16 isolates). The detection rates of heavy metal resistance genes were found to be generally higher in isolates from neritic-stage turtles compared to those from oceanic-stage turtles. Nevertheless, the arsenate reductase gene (*arsC*) exhibited a higher detection rate in oceanic isolates (Table 3, Table 4 and Table S2).

### 2.3. Associations Between Heavy Metal Resistance and Antimicrobial Resistance

When the association between the presence of specific heavy metal resistance genes (*arsC, chrA, merA, copA, and czcA*) and isolates with multiple antibiotic resistance (MAR) phenotypes (MAR and MARi > 0.20) was analysed, a significant association was found between isolates exhibiting MAR patterns and the presence of the mercuric reductase gene (*merA*) (*p* = 0.010; chi-squared test). Similarly, isolates with a MAR index greater than 0.20 exhibited a significantly higher prevalence of the merA gene (52.6%) compared to isolates with a MAR index < 0.20 (21.4%) (*p* = 0.027; chi-squared test) (Table 5).

## 3. Discussion

Although no significant differences in MAR index values were observed between neritic-stage and oceanic-stage groups (which may be attributed to the relatively limited sample size in our study), it was found that *Vibrio* spp. isolates with a multiple antimicrobial resistance (MAR) pattern and a MAR index value greater than 0.2 were more likely to be observed in neritic-stage sea turtles. Furthermore, isolates from neritic sea turtles were more resistant to most of the antimicrobial agents tested (except beta-lactams and macrolides) than isolates from oceanic sea turtles. It was also observed that isolates derived from neritic sea turtles exhibited greater resistance to nitrofurans (nitrofurantoin) and aminoglycosides (including amikacin and gentamicin) than those derived from oceanic sea turtles. We further found that 35% of isolates (all derived from neritic-stage turtles) were resistant to nitrofurans (nitrofurantoin) and aminoglycosides (including amikacin and gentamicin). Nevertheless, previous studies conducted over the past few decades have indicated that the majority of *Vibrio* spp. isolates were sensitive to nitrofurantoin, gentamicin, and amikacin. For example, Amaro et al. (1999) [66] isolated several *Vibrio vulnificus* strains that were sensitive to nitrofurantoin, amikacin, and gentamicin from aquatic habitats in Taiwan [66]. Moreover, Liu et al. (2004) [67] also reported that *Vibrio alginolyticus* isolates obtained from diseased white shrimp (*Litopenaeus vannamei*; also known as *Penaeus vannamei*) in Taiwanese culture ponds were all sensitive to nitrofurantoin [67]. In a retrospective analysis of 84 clinical case studies of patients with *Vibrio vulnificus* infection in Taiwan over a period of six years, from 1995 to 2000, Hsueh et al. (2004) [68] found that all isolates demonstrated susceptibility to gentamicin and amikacin. However, in recent decades, a significant number of bacteria, including *Vibrio*, have emerged with an unprecedented resistance to a wide range of antimicrobials. This could be a consequence of their misuse in both agricultural and human systems [20,62]. As reported by Baralla et al. (2021) [19], aminoglycosides and nitrofurans were used in aquaculture in China from 1996 to 2013. Nitrofurantoin was commonly employed in veterinary medicine as a treatment for protozoan and bacterial infections [31]. Aftabuddin et al. (2009) [38] reported the use of nitrofurans as a prophylactic agent for *Penaeus monodon* vibriosis in Bangladesh. Tonguthai (2000) [37] reported that nitrofurans were employed as a chemotherapeutic agent for the treatment of shrimp hatchery vibriosis in Thailand. Furthermore, amikacin and gentamicin are frequently employed in both human and veterinary medical industries to treat a spectrum of gram-negative bacterial infections [32,33,34]. The correlation between the emergence of aminoglycoside resistance and the extensive use of antimicrobials, particularly in the context of the prevention and treatment of *Vibrio cholerae*, as well as other applications, is well documented [20]. A study by Redpath et al. (2021) [69] has indicated that the veterinary use of aminoglycoside antimicrobials is currently under increasing scrutiny. In particular, gentamicin was commonly used empirically without bacterial culture and susceptibility testing [69]. Furthermore, the presence of *Vibrio* spp. that are non-susceptible to antimicrobial agents represents a significant concern in the context of plastic pollution. The presence of *Vibrio* spp. on microplastics has been demonstrated in a number of studies. These bacteria are found in high abundance on these particles [70,71]. In light of these observations, Canellas et al. (2021) [48] postulate that *Vibrio* spp. may be acquiring antimicrobial resistance genes (ARGs) from bacteria released into marine environments through sewage, and subsequently dispersed via plastic debris or in the form of plankton throughout the bay. This may have the potential to endanger the health of those exposed to this environment. A previous study by Kim et al. [33] indicates that a reduction in the consumption of aminoglycosides is associated with a reduction in the prevalence of resistance. This indicates the necessity of the implementation of an antimicrobial cycling strategy at the national level.

In this study, 48.9% of *Vibrio* species from sea turtles were found to have a MAR pattern, indicating resistance to at least three different classes of antimicrobials. Additionally, a statistically significant difference was observed between the nine *Vibrio* species and their respective MAR patterns (*p* = 0.033; Fisher’s exact test). The rank order of MAR patterns of the *Vibrio* spp. isolates evaluated was as follows: *V. rotifetianus* (1/1; 100.0%) > *V. harveyi* (7/9; 77.8%) > *V. vulnificus* (3/4; 75.0%) > *V. parahaemolyticus* (4/6; 66.7%) > *V. alginolyticus* (8/18; 44.4%) > *V. campbellii* (0/5; 0.0%), *V. fluvialis* (0/2; 0.0%), *V. mediterranei* (0/1; 0.0%), and *V. cyclitrophicus* (0/1; 0.0%) (Table 1). Among the 47 *Vibrio* isolates identified in this study, *V. alginolyticus* (18/47) and *V. harveyi* (9/47) were the most prevalent and second most prevalent species, respectively (Table 1). Among these, a total of eight out of 18 isolates of *V. alginolyticus* exhibited a MAR phenotype. *V. alginolyticus* is frequently found in sea turtles’ lesions [18,72,73]. Furthermore, *V. alginolyticus* also plays a multitude of roles in marine turtles. For instance, it is a constituent of the common flora in the oral cavity and cloaca of healthy sea turtles captured from foraging grounds [74]. Additionally, it has been identified as an opportunistic pathogen [75,76]. It has been linked to the development of dermatological conditions in an aquarium-reared loggerhead turtle [77]. The collective findings of the current and previous studies indicate that *V. alginolyticus* with MAR should be regarded as an important pathogen in sick and injured neritic-stage sea turtles in rehabilitation centres. With regard to public health, the species *V. alginolyticus*, *V. harveyi*, *V. parahaemolyticus*, and *V. vulnificus* are the major species often associated with human infections [24,78]. It is therefore recommended that veterinarians working in sea turtle rehabilitation facilities should be aware of the risk of exposure to these bacteria.

A total of 40.4% of all isolates in this study exhibited MAR index values exceeding 0.2, indicating a high risk of antimicrobial contamination. A MAR index score above 0.2 signifies a notable level of antimicrobial use in the area and predicts a high-risk environment for the spread of antimicrobial resistance. [24,30,79,80]. In this study, the MAR index exhibited a range between 0.00 and 0.73. This is a cause for concern, given that a study by Mohamad et al. (2019) [81] found that approximately 75% of *Vibrio* isolates from diseased fish in Malaysia also had a MAR index exceeding 0.2. Fernandes et al. (2021) [82] reported a low prevalence of multiple-antimicrobial-resistant gram-negative bacteria isolated from loggerhead sea turtles (*Caretta caretta*) in Cape Verde. Only two isolates (10%) with MAR index values greater than 0.2 were detected in that study: 0.25 for *E. cloacae* and 0.33 for *A. hydrophila/caviae*. We also found that the mean MAR index of *V. alginolyticus* identified in our study (ranging from 0.07 to 0.73) was significantly higher than that reported in wild loggerhead turtles (MAR index ranging from 0.00 to 0.08) [82]. Compared to studies by Sony et al. (2021) [24], who reported MAR indexes ranging from 0.05 to 0.47 in isolates from live diseased fishes, Ha et al. (2023) [30], who reported indexes ranging from 0.25 to 0.67 in farmed *Litopenaeus vannamei*, and Mohamad et al. (2019) [81], who reported indexes ranging from 0.06 to 0.56 in marine fishes, the results of our study imply a higher MAR index for *Vibrio* spp. in our study area.

Although our study found no significant difference in the MAR index between isolates from turtles found in different sea turtle stranding areas, the proportion of *Vibrio* spp. isolates with a MAR index greater than 0.2 in neritic-stage turtles (42.5% of isolates) was higher than the proportion found in oceanic-stage turtles (28.6% of isolates). These results suggest that sea turtles in neritic foraging habitats are at higher risk of antimicrobial contamination than those in pelagic environments [24,30,79,80,81,83]. As noted in a previous study, the number of oceanic-stage juveniles (curved carapace length < 30 cm) [59,61] identified in stranding reports in Taiwan is limited [1]. In other words, most of the sea turtle strandings found in Taiwan were neritic-stage individuals [84]. Indeed, Taiwan’s coastal waters are known to contain green turtle feeding and migration habitats [85,86,87]. Furthermore, several studies have reported that sea turtles are often exposed to antimicrobial residues and antimicrobial-resistant bacteria, which are common in polluted areas, due to their high frequency of coastal foraging, their long lifespans, and their frequent use of nearshore habitats affected by anthropogenic activities [6,11,15,16,17]. Although wild sea turtles are unlikely to have been exposed to antimicrobial therapy under natural conditions, resistance in bacteria isolated from wild sea turtles has become an increasing concern [5,6,13,14,58,82]. It is possible that the multiple-antimicrobial-resistant strains isolated from wild sea turtles are related to coastal wastewater pollution. Specifically, selection pressure may cause bacteria in marine habitats to develop resistance after exposure to antimicrobial-containing environmental effluents (e.g., agricultural, aquaculture, human, and veterinary waste effluents) [11,12,13,14,15,43,82,88,89,90,91,92].

Bacterial resistance to antimicrobials can also be influenced by other pollutants, such as heavy metals. For example, heavy metals may induce antimicrobial resistance in metal-contaminated environments, as previous studies have shown that heavy metals are correlated with the selection of antimicrobial resistance genes [41,42,43,44]. Although antimicrobial resistance genes can occur naturally in various environments in low abundance, studies have demonstrated that their abundance increases in the presence of several pollutants, including crude oil, sewage, and heavy metals [43,44,93,94]. In addition, the presence of heavy metal-resistant bacteria can indicate the degree of contamination in the environment and is frequently linked to the co-selection of antimicrobial resistance [48,95,96]. Mechanisms of antimicrobial and heavy metal resistance co-selection in environmental bacteria include cross-resistance, co-resistance, and co-regulation [97,98,99]. In our study, the rank order of the metal resistance gene detection rate of the *Vibrio* spp. isolates evaluated was as follows: *arsC* (70.2%; 33/47) > *chrA* (68.1%; 32/47) > *merA* (34.0%; 16/47) > *copA* (25.5%;12/47) > *czcA* (0.0%; 0/47). The analysis revealed a statistically significant differentiation between the nine *Vibrio* species and the rates of detection of their respective metal resistance genes, specifically *chrA*. (*p* = 0.021; Fisher’s exact test) and *copA* (*p* = 0.022; Fisher’s exact test) (Table 4). The most prevalent species with *chrA* and *copA*, respectively, were *V. parahaemolyticus* (6/6; 100.0%) and *V. vulnificus* (3/4; 75.0%) in our study. In addition, the presence of heavy metal resistance genes in isolates was more common in neritic-stage turtles than in oceanic-stage turtles (CCL < 30 cm), except for *arsC*, which was more common in oceanic-stage turtles. Fang et al. (2020) [46] reported in their study that 22.32% of *Vibrio parahaemolyticus* isolates identified from Pacific mackerel showed multi-heavy metal resistance (MHMR). A study conducted in southwest Nigeria showed that all *Vibrio* isolates from water in aquaculture ponds with high levels of metal contamination were able to tolerate five metals (copper, zinc, lead, nickel, and chromium) [47]. The heavy metal resistance gene (*copA*) has also been detected in *Vibrio* spp. isolates isolated from clams (*Meretrix meretrix*) in China [49]. Additionally, the majority of antimicrobial-resistant isolates in the same study showed tolerance to heavy metals, including Cd, Zn, and Cu [49]. A study conducted in China found that *V. parahaemolyticus* isolates from *Penaeus vannamei* at freshwater farms, seawater farms, and markets exhibited both multidrug resistance (MDR) and MHMR [41]. On the other hand, in a study conducted in Brazil, the detection of heavy metal resistance genes (*copA* and *merA*) was observed in antimicrobial-resistant *Vibrio* isolates [48]. In our study, a significant association was observed between MAR and the presence of *merA* (mercuric reductase gene) (*p* = 0.010). Similarly, a significant association was also observed between the presence of the *merA* gene and MAR index values greater than 0.2 (*p* = 0.027). In previous research on trace elements, methylmercury, and polybrominated diphenyl ethers in sea turtles in the South China region and Taiwan, liver methylmercury (*MeHg*) levels were 6–750 times higher than the range of *MeHg* levels observed in green turtles in Baja California, Mexico, and similar to those estimated for green turtles in the Mediterranean, a historically industrialised area [53]. Furthermore, a marginally significant association was also identified between the presence of the *chrA* gene and MAR index values exceeding 0.2 (*p* = 0.051) in our study. These findings suggest that heavy metal accumulation in the marine environment may induce bacterial resistance to these metals and co-select for resistance to antimicrobials [41,44,94,100].

## 4. Materials and Methods

### 4.1. Bacteria

Bacterial isolates (*n* = 47) belonging to nine different *Vibrio* spp., namely *V. alginolyticus* (*n* = 18), *V. campbellii* (*n* = 5), *V. harveyi* (*n* = 9), *V. parahaemolyticus* (*n* = 6), *V. vulnificus* (*n* = 4), *V. fluvialis* (*n* = 2), *V. mediterranei* (*n* = 1), *V. cyclitrophicus* (*n* = 1), and *V. rotifetianus* (*n* = 1) (Table 6), which were collected from the clinical work of veterinarians Dr. Tsung-Hsien Li and Dr. I-Chun Chen on rescued marine turtles (which had not received any antimicrobial therapy prior to the collection of the samples) brought to the sea turtle rehabilitation facility at the National Museum of Biology and Aquarium (NMMBA) in Checheng Township, Pingtung County, Taiwan for rehabilitation during 2017–2022, were used in the present study. All isolates were obtained from neritic-stage sea turtles (*n* = 40) and oceanic-stage sea turtles (*n* = 7) (Table 6). Therefore, no sea turtle individuals were specifically captured from the wild for the purposes of this study. All turtles were sourced from eastern Taiwan (*n* = 11) and southern Taiwan (*n* = 19). The turtles (*n* = 30; *Chelonia mydas*: 20, *Eretmochelys imbricate*: 5, *Lepidochelys olivacea*: 4, *Caretta caretta*: 1) were classified by life stage using reference values for each species (Table 6). For all species, turtles with a curved carapace length (CCL) of less than 30 cm were classified as oceanic-stage juveniles, while those with a CCL of 30 cm or greater were classified as neritic-stage juveniles [59,61]. The sample size in the present study is relatively limited. However, due to the endangered status of all sea turtles in Taiwan, no sea turtles were captured without the necessary permits.

The identification of these isolates (*Vibrio* spp.) was conducted in accordance with the methods described in our previous report. Briefly, *Vibrio* spp. isolates were verified by polymerase chain reaction using specific primers [6].

### 4.2. Determination of Antimicrobial Resistance and Heavy Metal Resistance Genes

The Kirby–Bauer disk diffusion method was employed on Mueller–Hinton agar (MHA; Difco, Sparks, USA) to assess antimicrobial resistance profiles. *Vibrio* strains were tested on MHA supplemented with 1% NaCl for optimal growth conditions. The selection of antimicrobial discs was based on a review of the existing literature on the subject of sea turtles and the *Vibrio* species. Eighteen different antimicrobial discs were used, mirroring prior published research [5,6,24,63,101,102]. These discs encompassed a broad spectrum of antibiotics including tetracyclines (doxycycline and oxytetracycline), nitrofurans (nitrofurantoin), folate pathway inhibitors (sulfamethoxazole/trimethoprim), cephalosporins (ceftriaxone, cefuroxime and ceftiofur), beta lactam–beta lactamase inhibitors (amoxicillin/clavulanic acid and amoxicillin), aminoglycosides (gentamicin and amikacin), fluoroquinolones (enrofloxacin and ciprofloxacin), phenicols (chloramphenicol), and macrolides (azithromycin) (Oxoid Ltd., Basingstoke, UK). Following inoculation with the bacterial isolates, plates were incubated at 28 °C for 24 h. Interpretation of inhibition zones followed the guidelines established by the Clinical and Laboratory Standards Institute (CLSI) [11,16,17,55,103,104]. Consistent with previous studies [13,64,104,105], isolates resistant to three or more antimicrobial categories were classified as exhibiting multiple antibiotic resistance (MAR).

The *Vibrio* strains were evaluated for the presence of five heavy metal resistance genes using primers and conditions given in the Table 7. The total PCR reaction volume was set as 25 μL, comprising 200 ng template DNA, 10 × PCR buffer, 10 mM dNTPs, 12.5 mΜ primers, and 0.25 U Taq DNA polymerase, and topped with DDW to 25 μL. The amplification conditions for the *arsC* and *chrA* genes in this study comprised an initial denaturation step at 94 °C for 5 min, followed by 35 cycles of denaturation at 95 °C for 30 s, annealing at 50 °C for 30 s and extension at 72 °C for 45 s, with the exception of the final cycle where the extension was for 7 min. The PCR products were checked by electrophoresis and visualized on a 2% agarose gel.

### 4.3. Data Analysis

The multiple antimicrobial resistance (MAR) index was determined for each isolated bacteria using the following formula: MAR index = A/B. Here, ‘A’ represents the number of antimicrobials to which the isolated bacteria showed resistance, and ‘B’ represents the total number of antimicrobials tested against the isolated bacteria. This method of calculation was described by Krumperman in 1983 [24,79]. The statistical significance of the observed differences between the groups was evaluated through the application of either the Mann–Whitney U test or the Kruskal–Wallis H test. The association between the categorical variables was analysed using the Fisher’s exact test/chi-squared test or the logistic regression model. All statistical analyses were performed by SPSS for Windows version 20.0 (Chicago, IL, USA).

## 5. Conclusions

To the best of our knowledge, this is the first study to present and compare the profiles of antimicrobial resistance, multiple antimicrobial resistance (MAR), MAR index, and heavy metal resistance genes of *Vibrio* spp. isolates from sea turtles of different life stages in Taiwan. *Vibrio* spp. isolates with a MAR pattern and a MAR index value exceeding 0.2 were more likely to be observed in neritic-stage sea turtles, suggesting that wild sea turtles may be exposed to polluted sewage containing antimicrobials and heavy metals when recruiting to nearshore habitats or foraging in coastal feeding areas. Furthermore, *Vibrio* isolates with MAR and MAR index values exceeding 0.2 were significantly more likely to harbour the *merA* gene. This suggests that the co-selection of antimicrobial resistance and heavy metal contamination may occur in inshore foraging habitats of sea turtles. Bacteria with antimicrobial resistance can limit therapeutic options and further complicate the effectiveness of treatment for rescued sea turtles with bacterial infections. However, current data on the pharmacokinetics of antimicrobials in sea turtles are very limited. Further studies are required to elucidate the pharmacokinetics of antimicrobials and confirm the optimal dosage regimen for different types of antibiotics in sea turtles. Moreover, future research could also examine a greater number of bacterial strains derived from a variety of sources, including agricultural, aquaculture, human, and veterinary waste effluents. This could facilitate the identification of potential contamination sources and drug-resistant situations.

## Figures and Tables

**Table 1:** Summary statistics of antimicrobial resistance in each species against the tested antimicrobial group/agents.

Antimicrobial Groups	*Vibrio* Species									*p*-Value
	*V. alginolyticus*	*V. harveyi*	*V. parahaemolyticus*	*V. campbellii*	*V. vulnificus*	*V. fluvialis*	*V. mediterranei*	*V. cyclitrophicus*	*V. rotifetianus*	
Tetracyclines	16.7% (3/18)	55.6% (5/9)	33.3% (2/6)	20.0% (1/5)	50.0% (2/4)	0.0% (0/2)	0.0% (0/1)	0.0% (0/1)	0.0% (0/1)	0.486
Nitrofurans	33.3% (6/18)	55.6% (5/9)	0.0% (0/6)	0.0% (0/5)	50.0% (2/4)	0.0% (0/2)	0.0% (0/1)	100.0% (1/1)	0.0% (0/1)	0.103
Folate pathway inhibitors	16.7% (3/18)	22.9% (2/9)	16.7% (1/6)	0.0% (0/5)	0.0% (0/4)	0.0% (0/2)	0.0% (0/1)	0.0% (0/1)	0.0% (0/1)	0.979
Cephalosporins	88.9% (16/18)	66.7% (6/9)	83.3% (5/6)	20.0% (1/5)	100.0% (4/4)	50.0% (1/2)	0.0% (0/1)	0.0% (0/1)	100.0% (1/1)	0.011
Beta lactam–beta lactamase inhibitors	88.9% (16/18)	66.7% (6/9)	83.3% (5/6)	60.0% (3/5)	100.0% (4/4)	100.0% (2/2)	0.0% (0/1)	0.0% (0/1)	100.0% (1/1)	0.153
Aminoglycosides	33.3% (6/18)	33.3% (3/9)	16.7% (1/6)	0.0% (0/5)	50.0% (2/4)	0.0% (0/2)	100.0% (1/1)	0.0% (0/1)	100.0% (1/1)	0.337
Fluoroquinolones	27.8% (5/18)	22.2% (2/9)	50.0% (3/6)	0.0% (0/5)	25.0% (1/4)	0.0% (0/2)	0.0% (0/1)	0.0% (0/1)	0.0% (0/1)	0.787
Phenicols	5.6% (1/18)	11.1% (1/9)	0.0% (0/6)	0.0% (0/5)	0.0% (0/4)	0.0% (0/2)	0.0% (0/1)	0.0% (0/1)	0.0% (0/1)	1.000
Macrolides	16.7% (3/18)	11.1% (1/9)	16.7% (1/6)	0.0% (0/5)	0.0% (0/4)	0.0% (0/2)	0.0% (0/1)	0.0% (0/1)	0.0% (0/1)	1.000
MAR ^a^	44.4% (8/18)	77.8% (7/9)	66.7% (4/6)	0.0% (0/5)	75.0% (3/4)	0.0% (0/2)	0.0% (0/1)	0.0% (0/1)	100.0% (1/1)	0.033

^a^ The MAR was defined as isolates that exhibited resistance to at least one agent in three or more antimicrobial categories.

**Table 2:** Summary of antimicrobial resistance phenotypic patterns in all *Vibrio* spp. isolates (*n* = 47) and the resistance percentage in isolates from sea turtles at the neritic and oceanic stages.

Antimicrobial Groups	Life Stage		
	Neritic Stage	Oceanic Stage	*p*-Value
	Percentage of Resistant Isolates	Percentage of Resistant Isolates	
Tetracyclines	30.0% (12/40)	14.3% (1/7)	0.655
Nitrofurans	35.0% (14/40)	0.0% (0/7)	0.086
Folate pathway inhibitors	15.0% (6/40)	0.0% (0/7)	0.571
Cephalosporins	75.0% (30/40)	57.1% (4/7)	0.377
Beta lactam–beta lactamase inhibitors	75.0% (30/40)	100.0% (7/7)	0.318
Aminoglycosides	35.0% (14/40)	0.0% (0/7)	0.086
Fluoroquinolones	25.0% (10/40)	14.3% (1/7)	1.000
Phenicols	5.0% (2/40)	0.0% (0/7)	1.000
Macrolides	10.0% (4/40)	14.3% (1/7)	0.571

**Table 3:** Results for the isolates were assessed via polymerase chain reaction (PCR) to identify heavy metal resistance genes and compared across different life stages of sea turtles.

Metal Resistance Genes	Life Stage		*p*-Value
	Neritic Stage	Oceanic Stage	
Copper resistance gene (*copA*)	27.5% (11/40)	14.3% (1/7)	0.659
Cadmium resistance gene (*czcA*)	0.0% (0/40)	0.0% (0/7)	-
Mercuric reductase gene (*merA*)	37.5% (15/40)	14.3% (1/7)	0.396
Arsenate reductase gene (*arsC*)	67.5% (27/40)	85.7% (6/7)	0.657
Chromium resistance gene (*chrA*)	70.0% (28/40)	57.1% (4/7)	0.664

**Table 4:** Summary statistics of carrying various metal resistance genes in each *Vibrio* species.

Metal Resistance Genes	*Vibrio* Species									*p*-Value
	*V. alginolyticus*	*V. harveyi*	*V. parahaemolyticus*	*V. campbellii*	*V. vulnificus*	*V. fluvialis*	*V. mediterranei*	*V. cyclitrophicus*	*V. rotifetianus*	
Copper resistance gene (*copA*)	11.1% (2/18)	22.2% (2/9)	66.7% (4/6)	0.0% (0/5)	75.0% (3/4)	50.0% (1/2)	0.0% (0/1)	0.0% (0/1)	0.0% (0/1)	0.022
Cadmium resistance gene (*czcA*)	0.0% (0/18)	0.0% (0/9)	0.0% (0/6)	0.0% (0/5)	0.0% (0/4)	0.0% (0/2)	0.0% (0/1)	0.0% (0/1)	0.0% (0/1)	-
Mercuric reductase gene (*merA*)	38.9% (7/18)	33.3% (3/9)	33.3% (2/6)	40.0% (2/5)	25.0% (1/4)	50.0% (1/2)	0.0% (0/1)	0.0% (0/1)	0.0% (0/1)	1.000
Arsenate reductase gene (*arsC*)	77.8% (14/18)	55.6% (5/9)	66.7% (4/6)	80.0% (4/5)	50.0% (2/4)	100.0% (2/2)	100.0% (1/1)	0.0% (0/1)	100.0% (1/1)	0.678
Chromium resistance gene (*chrA*)	83.3% (15/18)	66.7% (6/9)	100.0% (6/6)	60.0% (3/5)	25.0% (1/4)	50.0% (1/2)	0.0% (0/1)	0.0% (0/1)	0.0% (0/1)	0.021

**Table 5:** The associations between the antimicrobial resistance phenotypes and the metal resistance genes detected in the isolates.

Metal Resistance Genes	Multiple Antimicrobial Resistance (MAR) ^a^		*p*-Value	MAR Index ^b^		*p*-Value
	MAR	Non-MAR		MAR Index > 0.2	MAR Index ≤ 0.2	
Copper resistance gene (*copA*)	26.1% (6/23)	25.0% (6/24)	0.932	21.1% (4/19)	28.6% (8/28)	0.737
Cadmium resistance gene (*czcA*)	0.0% (0/23)	0.0% (0/24)	-	0.0% (0/19)	0.0% (0/28)	-
Mercuric reductase gene (*merA*)	52.2% (12/23)	16.7% (4/24)	0.010	52.6% (10/19)	21.4% (6/28)	0.027
Arsenate reductase gene (*arsC*)	78.3% (18/23)	62.5% (15/24)	0.238	78.9% (15/19)	64.3% (18/28)	0.281
Chromium resistance gene (*chrA*)	78.3% (18/23)	58.3% (14/24)	0.143	84.2% (16/19)	57.1% (16/28)	0.051

^a^ The MAR was defined as isolates that exhibited resistance to at least one agent in three or more antimicrobial categories. ^b^ The multiple antimicrobial resistance (MAR) index.

**Table 6:** Details of *Vibrio* isolates used in the study.

Turtle Number	Species	Geographical Location	Life Stage	*Vibrio* Species
1	*C. mydas*	Southern Taiwan	Neritic stage	*V. harveyi*
2	*C. mydas*	Eastern Taiwan	Neritic stage	*V.vulnificus*
3	*C. mydas*	Eastern Taiwan	Neritic stage	*V.alginolyticus*
4	*C. mydas*	Eastern Taiwan	Neritic stage	*V.parahaemolyticus*
5	*C. mydas*	Eastern Taiwan	Neritic stage	*V.campbellii*
6	*C. mydas*	Southern Taiwan	Neritic stage	*V.parahaemolyticus*
7	*C. mydas*	Southern Taiwan	Neritic stage	*V.parahaemolyticus*
8	*C. mydas*	Southern Taiwan	Neritic stage	*V.alginolyticus*
				*V.vulnificus*
				*V.alginolyticus*
				*V.alginolyticus*
9	*C. mydas*	Southern Taiwan	Neritic stage	*V.harveyi*
10	*C. mydas*	Southern Taiwan	Neritic stage	*V.alginolyticus*
11	*C. mydas*	Eastern Taiwan	Neritic stage	*V.harveyi*
12	*C. mydas*	Southern Taiwan	Neritic stage	*V.alginolyticus*
				*V.alginolyticus*
13	*C. mydas*	Southern Taiwan	Neritic stage	*V.parahaemolyticus*
14	*C. mydas*	Southern Taiwan	Neritic stage	*V.alginolyticus*
15	*C. mydas*	Southern Taiwan	Neritic stage	*V.harveyi*
16	*C. mydas*	Eastern Taiwan	Neritic stage	*V.alginolyticus*
				*V.alginolyticus*
17	*C. mydas*	Southern Taiwan	Neritic stage	*V. cyclitrophicus*
18	*C. mydas*	Southern Taiwan	Neritic stage	*V.alginolyticus*
				*V.harveyi*
19	*C. mydas*	Southern Taiwan	Oceanic stage	*V.campbellii*
				*V.campbellii*
				*V.campbellii*
20	*C. mydas*	Southern Taiwan	Neritic stage	*V. rotifetianus*
1	*E. imbricate*	Southern Taiwan	Oceanic stage	*V.harveyi*
2	*E. imbricate*	Southern Taiwan	Oceanic stage	*V.parahaemolyticus*
3	*E. imbricate*	Eastern Taiwan	Neritic stage	*V.vulnificus*
3	*E. imbricate*	Eastern Taiwan	Neritic stage	*V.harveyi*
4	*E. imbricate*	Southern Taiwan	Neritic stage	*V.harveyi*
				*V.campbellii*
5	*E. imbricate*	Southern Taiwan	Neritic stage	*V.harveyi*
				*V.alginolyticus*
1	*L. olivacea*	Eastern Taiwan	Neritic stage	*V. fluvialis*
				*V.parahaemolyticus*
2	*L. olivacea*	Eastern Taiwan	Oceanic stage	*V.alginolyticus*
				*V. fluvialis*
3	*L. olivacea*	Southern Taiwan	Neritic stage	*V.alginolyticus*
				*V.alginolyticus*
				*V. mediterranei*
				*V.alginolyticus*
4	*L. olivacea*	Eastern Taiwan	Neritic stage	*V.alginolyticus*
				*V.alginolyticus*
1	*C. caretta*	Eastern Taiwan	Neritic stage	*V.vulnificus*

**Table 7:** The target genes and PCR primers used in this research to investigate heavy metal resistance.

Analysed Gene	Primer	Primer Sequence (5′–3′)	Annealing Temperature	Product Size	Reference
Copper resistance gene (*copA*)	copA-F	CGGTCTCTACGAATACCGCTTCAA	55 °C	1300 bp	[45]
	copA-R	GAAATAGCTCATTGCCGAGGCGTT			
Cadmium resistance gene (*czcA*)	czcA-F	GTTCACCTTGCTCTTCGCCATGTT	55 °C	320 bp	
	czcA-R	ACAGGTTGCGGATGAAGGAGATCA			
Mercuric reductase gene (*merA*)	merA-F	GTGCCGTCCAAGATCATGAT	57 °C	933 bp	
	merA-R	TAGCCYACRGTSGCSACYTG			
Arsenate reductase gene (*arsC*)	V-ArsC-F	CAAAATGGYGTAWCACCGGAA	50 °C	186 bp	This study
	V-ArsC-R	YGCTTCGAACAGYTGMTCAT			
Chromium resistance gene (*chrA*)	V-chrA-F	TGATCATCATGTTGGCGCTG	50 °C	480 bp	This study
	V-chrA-R	CYTCTTGGCTGAGYTGYTCG			

## Data Availability

Data are contained within the article or Supplementary Material.

## Supplementary Materials

The following supporting information can be downloaded at: https://www.mdpi.com/article/10.3390/antibiotics13110998/s1, Table S1: Distribution of antimicrobial resistance patterns among *Vibrio* spp. isolates from sea turtles; Table S2: Heavy metal resistance characteristics of *Vibrio* spp. isolated from sea turtles.
