# Supplementary material for: Further Evidence of Anthropogenic Impact: High Levels of Multiple-Antimicrobial-Resistant Bacteria Found in Neritic-Stage Sea Turtles
Source: Antibiotics (Basel). 2024 Oct 22;13(11):998. doi: 10.3390/antibiotics13110998 (PMC11591244; doi:10.3390/antibiotics13110998)
Supplement: Supplementary file 1 [file antibiotics-13-00998-s001.zip › antibiotics-3224683-supplementary.pdf]

Supplementary Table S1. Distribution of antimicrobial resistance patterns among *Vibrio* spp. isolates from sea turtles.

Vibrio species	Susceptibility-resistance cut-off values [b]	Antimicrobial agents [a]														
		T30	XNL30	F/M300	D30	SXT	CRO30	AN30	ENO5	GM10	AZM15	CXM30	AMX25	CIP5	C30	AMC
	R	≦15	≦17	≦14	≦12	≦10	≦13	≦14	≦16	≦12	≦13	≦14	≦15	≦15	≦12	≦13
	I	16~25	18~20	15~16	13~15	11~15	14~20	15~16	17~20	13~14	14~17	15~17	16~20	16~20	13~17	14~17
	S	≧26	≧21	≧17	≧16	≧16	≧21	≧17	≧21	≧15	≧18	≧18	≧25	≧21	≧18	≧18
V.harveyi		15	11	22	23	20	12	16	24	15	16	0	0	24	28	0
V.harveyi		20	18	20	20	20	16	17	18	16	14	18	17	21	22	20
V.vulnificus		26	21	22	25	30	18	16	22	17	17	12	14	24	28	18
V.alginolyticus		26	14	25	23	25	22	18	26	19	18	12	12	25	29	20
V.parahaemolyticus		12	17	20	20	23	12	16	14	16	17	14	0	21	28	15
V.parahaemolyticus		19	21	20	21	24	0	21	0	21	11	19	0	14	22	0
V.campbellii		22	26	24	12	28	20	23	25	23	24	20	24	28	26	30
V.vulnificus		24	0	22	10	28	22	24	26	22	33	14	0	24	20	13
V.harveyi		40	38	13	12	10	22	22	32	22	28	22	0	34	44	30
V.parahaemolyticus		24	26	10	22	26	0	22	31	20	17	19	19	36	30	15
V.parahaemolyticus		16	24	22	10	0	19	17	18	15	16	0	0	20	20	10
V.alginolyticus		15	18	20	22	28	10	21	24	18	18	0	18	21	24	22
V.vulnificus		12	0	14	24	25	12	13	0	11	15	0	0	10	18	0
V.harveyi		14	18	22	20	22	16	14	0	16	0	16	16	18	20	18
V.alginolyticus		20	21	24	21	24	22	22	12	20	22	19	18	25	26	20
V.alginolyticus		13	19	12	15	0	0	0	0	0	0	0	0	16	18	0
V.alginolyticus		20	0	0	24	25	0	0	17	0	14	0	0	0	16	0
V.harveyi		25	0	14	26	23	0	0	0	0	15	0	0	0	18	0
V.alginolyticus		26	26	13	26	10	20	19	17	14	10	13	0	10	11	12
V.alginolyticus		19	0	0	22	0	18	0	18	0	16	0	0	16	20	11
V.parahaemolyticus		21	24	23	23	25	22	13	10	13	17	15	16	18	22	11
V.harveyi		0	0	0	0	17	21	0	18	15	18	16	0	16	20	0

V.campbellii	22	25	26	21	22	24	20	24	18	19	18	19	2	30	22
V.alginolyticus	26	21	20	28	27	16	12	18	0	0	0	0	17	19	0
V.harveyi	25	24	21	11	0	15	16	17	19	20	0	0	17	0	0
V. fluvialis	19	22	18	22	23	23	19	20	21	18	12	10	19	19	12
V.parahaemolyticus	21	21	23	22	26	22	21	22	20	19	14	10	22	25	22
V.alginolyticus	28	30	26	30	30	20	22	26	24	22	20	14	30	32	13
V.alginolyticus	30	29	26	29	31	20	22	25	23	24	13	14	22	28	13
V.alginolyticus	24	23	18	26	24	19	19	20	19	20	14	0	19	26	0
V. fluvialis	26	26	25	25	26	21	23	20	17	22	20	25	24	26	12
V.alginolyticus	19	25	22	21	23	18	19	19	16	18	0	0	19	22	0
V.alginolyticus	20	22	19	21	19	22	20	21	18	18	0	0	22	26	0
V. mediterranei	16	22	22	17	25	18	14	18	12	15	15	16	18	19	17
V.alginolyticus	18	21	19	22	21	18	16	21	16	18	12	0	20	22	0
V.harveyi	22	0	13	21	18	16	15	17	17	19	0	0	21	23	16
V.alginolyticus	26	20	22	24	22	22	19	21	20	22	19	12	24	27	19
V. cyclitrophicus	28	26	14	26	17	21	18	20	14	16	16	17	20	26	20
V.vulnificus	24	19	20	26	22	16	0	19	18	17	0	0	21	20	17
V.alginolyticus	23	23	12	22	25	17	16	17	18	16	14	0	22	22	18
V.harveyi	25	23	20	24	24	20	18	24	19	21	20	17	21	26	22
V.alginolyticus	13	0	26	24	26	0	0	18	18	17	0	0	18	26	22
V.alginolyticus	23	0	13	23	28	22	13	0	0	15	0	0	18	30	25
V.campbellii	26	24	24	26	23	22	20	21	19	20	20	14	23	29	21
V.campbellii	24	0	17	25	19	20	19	20	16	19	14	13	20	26	19
V.campbellii	26	25	21	26	26	22	19	20	18	23	20	14	22	30	20
V. rotifetianus	25	28	24	28	26	19	14	18	17	20	14	10	24	28	19

[a] T30: oxytetracycline; XNL30: ceftiofur; F/M300: nitrofurantoin; D30: doxycycline ;SXT: sulfamethoxazole/trimethoprim; CRO30: ceftriaxone; AN30: amikacin; ENO5: enrofloxacin; GM10: gentamicin; AZM15: azithromycin; CXM30: cefuroxime; AMX25: amoxicillin; CIP5: ciprofloxacin; C30: chloramphenicol; AMC: amoxicillin/clavulanic acid. [b] R: resistant; I: intermediate; S: susceptible.

Supplementary Table S2. Heavy metal resistance characteristics of *Vibrio* spp. isolated from sea turtles.

Strains	*copA*	*czcA*	*merA*	*arsC*	*chrA*
V.harveyi	-	-	-	-	-
V.harveyi	+	-	-	-	+
V.vulnificus	-	-	-	-	-
V.alginolyticus	+	-	-	-	+
V.parahaemolyticus	+	-	-	+	+
V.parahaemolyticus	-	-	-	+	+
V.campbellii	-	-	-	-	-
V.vulnificus	+	-	+	-	-
V.harveyi	+	-	+	-	+
V.parahaemolyticus	+	-	+	-	+
V.parahaemolyticus	-	-	-	+	+
V.alginolyticus	-	-	-	+	-
V.vulnificus	+	-	-	+	+
V.harveyi	-	-	-	-	-
V.alginolyticus	-	-	-	-	+
V.alginolyticus	-	-	+	+	+
V.alginolyticus	-	-	+	+	+
V.harveyi	-	-	-	+	+
V.alginolyticus	-	-	+	+	+
V.alginolyticus	-	-	+	+	+
V.parahaemolyticus	+	-	+	-	+
V.harveyi	-	-	+	+	+
V.campbellii	-	-	+	+	+
V.alginolyticus	-	-	+	+	+
V.harveyi	-	-	+	+	+

V. fluvialis	-	-	+	+	+
V.parahaemolyticus	+	-	-	+	+
V.alginolyticus	-	-	-	+	+
V.alginolyticus	-	-	-	+	-
V.alginolyticus	-	-	-	+	+
V. fluvialis	+	-	-	+	-
V.alginolyticus	-	-	-	-	+
V.alginolyticus	-	-	-	+	+
V. mediterranei	-	-	-	+	-
V.alginolyticus	-	-	-	-	-
V.harveyi	-	-	-	+	+
V.alginolyticus	+	-	-	+	+
V. cyclitrophicus	-	-	-	-	-
V.vulnificus	+	-	-	+	-
V.alginolyticus	-	-	+	+	+
V.harveyi	-	-	-	+	-
V.alginolyticus	-	-	+	+	+
V.alginolyticus	-	-	-	+	+
V.campbellii	-	-	+	+	-
V.campbellii	-	-	-	+	+
V.campbellii	-	-	-	+	+
V. rotifetianus	-	-	-	+	-

⁺ Indicates that the isolate with heavy metal resistance genes in the table.
